# Opaganib (ABC294640) Induces Immunogenic Tumor Cell Death and Enhances Checkpoint Antibody Therapy

**DOI:** 10.3390/ijms242316901

**Published:** 2023-11-29

**Authors:** Lynn W. Maines, Staci N. Keller, Charles D. Smith

**Affiliations:** Apogee Biotechnology Corporation, 1214 Research Blvd, Suite 2015, Hummelstown, PA 17036, USA; lwmaines@apogee-biotech.com (L.W.M.);

**Keywords:** opaganib, ABC294640, sphingolipid, immunogenic cell death, immunotherapy, checkpoint antibody

## Abstract

Antibody-based cancer drugs that target the checkpoint proteins CTLA-4, PD-1 and PD-L1 provide marked improvement in some patients with deadly diseases such as lung cancer and melanoma. However, most patients are either unresponsive or relapse following an initial response, underscoring the need for further improvement in immunotherapy. Certain drugs induce immunogenic cell death (ICD) in tumor cells in which the dying cells promote immunologic responses in the host that may enhance the in vivo activity of checkpoint antibodies. Sphingolipid metabolism is a key pathway in cancer biology, in which ceramides and sphingosine 1-phosphate (S1P) regulate tumor cell death, proliferation and drug resistance, as well as host inflammation and immunity. In particular, sphingosine kinases are key sites for manipulation of the ceramide/S1P balance that regulates tumor cell proliferation and sensitivity to radiation and chemotherapy. We and others have demonstrated that inhibition of sphingosine kinase-2 by the small-molecule investigational drug opaganib (formerly ABC294640) kills tumor cells and increases their sensitivities to other drugs and radiation. Because sphingolipids have been shown to regulate ICD, opaganib may induce ICD and improve the efficacy of checkpoint antibodies for cancer therapy. This was demonstrated by showing that in vitro treatment with opaganib increases the surface expression of the ICD marker calreticulin on a variety of tumor cell types. In vivo confirmation was achieved using the gold standard immunization assay in which B16 melanoma, Lewis lung carcinoma (LLC) or Neuro-2a neuroblastoma cells were treated with opaganib in vitro and then injected subcutaneously into syngeneic mice, followed by implantation of untreated tumor cells 7 days later. In all cases, immunization with opaganib-treated cells strongly suppressed the growth of subsequently injected tumor cells. Interestingly, opaganib treatment induced crossover immunity in that opaganib-treated B16 cells suppressed the growth of both untreated B16 and LLC cells and opaganib-treated LLC cells inhibited the growth of both untreated LLC and B16 cells. Next, the effects of opaganib in combination with a checkpoint antibody on tumor growth in vivo were assessed. Opaganib and anti-PD-1 antibody each slowed the growth of B16 tumors and improved mouse survival, while the combination of opaganib plus anti-PD-1 strongly suppressed tumor growth and improved survival (*p* < 0.0001). Individually, opaganib and anti-CTLA-4 antibody had modest effects on the growth of LLC tumors and mouse survival, whereas the combination of opaganib with anti-CTLA-4 substantially inhibited tumor growth and increased survival (*p* < 0.001). Finally, the survival of mice bearing B16 tumors was only marginally improved by opaganib or anti-PD-L1 antibody alone but was nearly doubled by the drugs in combination (*p* < 0.005). Overall, these studies demonstrate the ability of opaganib to induce ICD in tumor cells, which improves the antitumor activity of checkpoint antibodies.

## 1. Introduction

Immunotherapy provides important benefits as first- or second-line therapy for several cancers including melanoma and lung cancer. In particular, antibody-based therapies targeting CTLA-4 and the PD-1/PD-L1 checkpoint pathways have reached FDA approval because of improved patient survival; however, the majority of cancer patients do not respond to checkpoint antibodies, and in the case of melanoma have a durable complete response rate of only ~15% [1,2]. Clearly, these new drugs are exciting advances in cancer therapeutics; however, much work is currently in progress to identify rational combinations of checkpoint antibodies with other drugs or radiation [3,4,5]. Cancer cells that are dying because of exposure to specific drugs or radiation can elicit an innate immune response through a process termed immunogenic cell death (ICD). For example, cancer cells exposed to doxorubicin, mitoxantrone, oxaliplatin or bortezomib undergo a discrete form of cell death, i.e., ICD, that generates adjuvants that promote antitumor immunity in immunocompetent mice [6,7,8]. ICD is indicated by a hallmark signature that involves induction of endoplasmic reticulum (ER) stress, followed by eIF2A phosphorylation-dependent exposure of normally ER resident proteins such as calreticulin on the plasma membrane of the dying cancer cells. ICD also involves the autophagy-mediated secretion of ATP, and the extracellular release of the high-mobility group box 1 (HMGB1) protein. Intriguingly, several studies have demonstrated that agents that induce ICD improve the antitumor activity of the antibodies (reviewed in [9,10]). This enhanced efficacy can be seen with antibodies directed against CTLA-4, PD-1 and/or PDL1 [10,11,12,13], providing an opportunity for an effective ICD inducer to be broadly combined with FDA-approved immunotherapy drugs.

Sphingolipid metabolism is being increasingly recognized as a key pathway in cancer biology in which ceramides, dihydroceramides (dhCer), sphingosine and sphingosine 1-phosphate (S1P) regulate tumor cell death, proliferation and drug resistance, as well as host angiogenesis, inflammation and immunity (reviewed in [14,15,16,17]). In particular, sphingosine kinases (SK1 and SK2) are key sites for manipulation of the ceramide/S1P rheostat that regulates tumor cell proliferation and death, as well as tumor sensitivity to radiation and chemotherapy (reviewed in [18,19,20]). We and others have demonstrated that SKs are frequently overexpressed in many cancers, and that inhibition of SK kills tumor cells [15]. In parallel, dhCer desaturase (DES1) controls the balance between saturated and unsaturated ceramides and this regulates proliferative and autophagic signaling in cancer cells. Sphingolipid metabolism has been recently considered as a potential modulator of cancer immunotherapy including approaches using vaccines, monoclonal antibodies, interleukins, checkpoint inhibitors and chimeric antigen receptor T cells [21]. Therefore, pharmacologic manipulation of sphingolipids may improve clinical responses to cancer immunotherapy.

Opaganib is an orally active, isozyme-selective inhibitor of SK2, and is competitive with respect to sphingosine [22,23]. Opaganib depletes S1P and elevates ceramide in tumor cells, suppresses signaling through pERK, pAKT and NFκB, and promotes autophagy and/or apoptosis [22,23,24,25,26]. Opaganib also downregulates c-Myc in a variety of cell lines [26,27,28,29]. Because it acts as a sphingosine mimetic, opaganib also inhibits DES1, increasing levels of dihydroceramides [27] and promoting autophagy in those cells and glucosylceramide synthase (GCS) reducing levels of glucosylceramide. Opaganib has antitumor activity in a wide range of mouse models as a single agent or in combination with other anticancer drugs [22,26,27,28,30,31,32,33,34]. A Phase 1 trial conducted with patients with advanced solid tumors demonstrated that opaganib is well-tolerated when administered orally on a twice-daily (BID) continuous schedule [35]. Overall, 64% of patients who completed 2 cycles of opaganib treatment had stable disease or better, suggesting that it has antitumor activity in most patients. In a second clinical trial of opaganib, 58% of patients with refractory multiple myeloma achieved stable disease or better, and patients had decreased plasma levels of TNFα, EGF and VEGF [36]. Opaganib is currently in Phase 2 clinical testing in patients having cholangiocarcinoma or prostate cancer.

The present studies focus on in vivo evaluation of the ability of opaganib to promote ICD in tumor cells and the antitumor activity of combinations of opaganib with a checkpoint antibody. Together, they provide support for the clinical testing of these combinations in cancer patients.

## 2. Results

Increased cell surface expression of calreticulin has been used as an in vitro marker of ICD in response to a variety of agents, including opaganib in combination with methotrexate- [37] or ABT-263/AZD-5991-treated [38] colorectal cells. To expand these studies, we measured the effects of in vitro opaganib treatment on the expression of calreticulin in a panel of diverse tumor cell lines. As shown in Table 1, opaganib increased calreticulin cell surface expression on all the cancer cells tested, with log responses ranging from 1.46 to 3.64, corresponding to ~3-fold to >400-fold increases in the pancreas, prostate, neuroblastoma, breast, lung and melanoma tumor cell lines. We have previously demonstrated that in vivo treatment of tumor-bearing mice with 100 mg/kg opaganib results in intratumor amounts of drug >100 µM for at least 5 h [22]. Therefore, the opaganib concentrations achievable in vivo can elevate expression of this key biochemical indicator of ICD.

**Figure 1:**
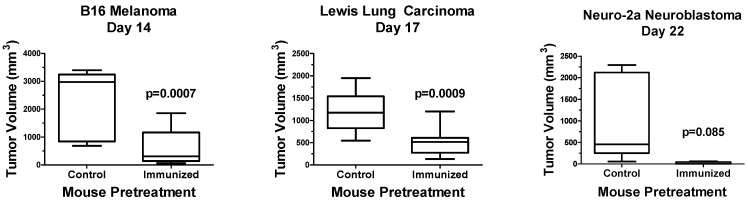
Opaganib promotes ICD in multiple tumor types. B16 melanoma, Lewis lung carcinoma or Neuro-2a neuroblastoma cells were treated in vitro with 40 μM opaganib for 24 h and then injected subcutaneously into C57BL/6 (B16 and LLC) or A/J (Neuro-2a) mice. Control mice received subcutaneous injection of PBS. After 7 days, all mice were injected with 1/5 the number of untreated matched cells, and tumor growth was monitored until tumors reached ≥3000 mm^3^. N = 10/group for B16 and LLC tumors and N = 5/group for Neuro-2a tumors. The median tumor volume is indicated by the horizontal line in each bar; the range of the bar indicates the interquartile range; and the whiskers indicate the range between the smallest and largest tumors for each treatment group.

To expand these studies, opaganib was evaluated using the gold standard in vivo vaccination assay to confirm ICD [39]. Murine melanoma B16, LLC or Neuro-2a neuroblastoma cells were treated in vitro with 40 μM opaganib for 24 h and then were implanted subcutaneously into immunocompetent mice as detailed in the Methods section. Control groups were injected in the left hind flank with phosphate-buffered saline (PBS) alone. After 7 days, mice in both groups were implanted with 1/5 the number of untreated matching tumor cells on the right hind flank, and tumor growth was measured until mice were euthanized when tumor volumes reached ≥ 3000 mm^3^. As shown in Figure 1, B16 tumor sizes on Day 14 after implantation into PBS-pretreated mice (control) and mice pretreated with opaganib-treated B16 cells (immunized) were 2344 ± 361 mm^3^ and 641 ± 210 mm^3^, respectively (*p* = 0.0007). LLC tumor sizes on Day 17 after implantation into PBS-pretreated mice (control) and mice pretreated with opaganib-treated LLC cells (immunized) were 1190 ± 143 and 510 ± 94 mm^3^, respectively (*p* = 0.0009). Neuro-2a tumor sizes on Day 22 after implantation into PBS-pretreated mice (control) and mice pretreated with opaganib-treated Neuro-2a cells (immunized) were 1039 ± 450 mm^3^ and 15 ± 15 mm^3^, respectively (*p* = 0.085). Whereas all of the mice in the control group had tumors, 75% of the Neuro-2a immunized mice were without measurable tumors on Day 22. Overall, these data demonstrate that treatment of tumor cells with opaganib induces ICD, which markedly reduces tumor growth in subsequently challenged mice.

**Figure 2:**
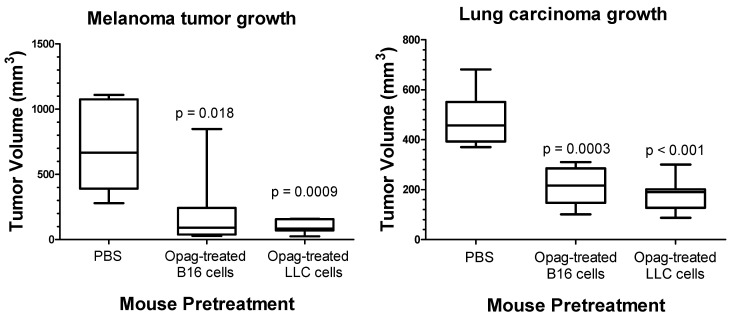
Opaganib promotes crossover immunity against tumors. (**Left panel**): Administration of opaganib-treated B16 melanoma or Lewis lung carcinoma (LLC) cells elicits immunity against subsequently injected untreated B16 tumor cells. (**Right panel**): Administration of opaganib-treated B16 melanoma or (LLC) cells elicits immunity against subsequently injected untreated LLC tumor cells.

We also tested the hypothesis that administration of one type of opaganib-treated tumor cells suppresses growth of not only the same type of tumor cell, but also provides crossover immunity to different types of tumor cells. Separately, B16 or LLC cells were treated in culture with 40 µM opaganib for 24 h to induce ICD. Opaganib-treated B16 or LLC cells were then harvested and injected (500,000 dying B16 cells or 5,000,000 dying LLC cells) in the left hind flank subcutaneously in 0.1 mL total volume of PBS. Control mice were injected in the left hind flank with PBS alone. After 7 days, mice were randomized into 4 groups and were challenged with either 100,000 live B16 cells or 1,000,000 live LLC cells on the right hind flank to evaluate tumor growth. Thus, the crossover test groups were mice immunized with opaganib-treated lung carcinoma cells and challenged with untreated melanoma cells; and mice immunized with opaganib-treated melanoma cells and challenged with untreated lung carcinoma cells. Tumor growth was measured, and mice were euthanized when tumor volumes reached ≥3000 mm^3^. Figure 2 shows data for B16 tumor size on Day 19 after implantation into either PBS-pretreated mice (control), mice pretreated with opaganib-treated B16 cells or mice pretreated with opaganib-treated LLC cells. B16 tumors in the control mice reached an average size of 702 ± 144 mm^3^. In contrast, cells injected into the B16 immunized mice reached an average size of 203 ± 15 mm^3^ (*p* = 0.018); while cells injected into the LLC immunized mice reached an average size of 102 ± 51 mm^3^ (*p* = 0.0009). Thus, vaccination with either opaganib-treated melanoma or lung carcinoma cells suppressed the subsequent growth of untreated melanoma cells. Figure 2 also shows data for LLC tumor size on Day 28 after implantation into either PBS-pretreated mice (control), mice pretreated with opaganib-treated B16 cells or mice pretreated with opaganib-treated LLC cells. Lung tumors in the control mice reached an average size of 479 ± 113 mm^3^. In contrast, cells injected into the B16 immunized mice reached an average size of 208 ± 74 mm^3^ (*p* = 0.0003); while cells injected into the LLC immunized mice reached an average size of 177 ± 68 mm^3^ (*p* < 0.001). Thus, vaccination with either opaganib-treated melanoma or lung carcinoma cells suppressed the subsequent growth of untreated lung carcinoma cells. These data demonstrate that in vitro treatment of tumor cells with opaganib promotes immunity to multiple tumor types in subsequently challenged mice.

### Effects of Combination of Opaganib and Checkpoint Antibodies on Tumor Growth

**Figure 3:**
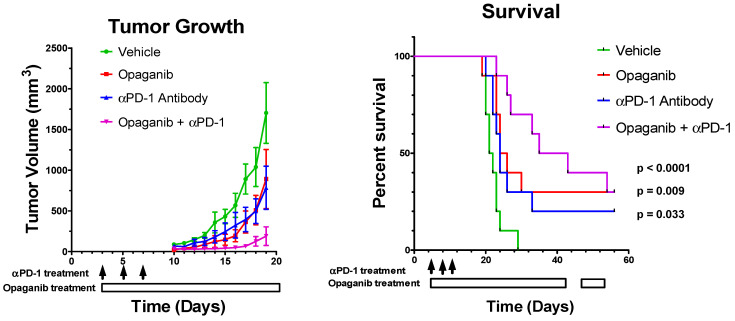
Antitumor activity of opaganib in combination with anti-PD-1 antibody. B16 cells were grown as xenografts and treated with the vehicle; opaganib alone (50 mg/kg/day, 5 days/week); anti-PD-1 antibody (200 μg/mouse intraperitoneally, 3 times as indicated by the black arrows); or opaganib+anti-PD-1. (**Left panel**): The mean ± SD tumor volume for each treatment group (n = 10) is shown. (**Right panel**): Individual mice were sacrificed when tumors exceeded 3000 mm^3^ and survival for each group is shown.

The combined effects of treating tumor-bearing mice with opaganib and anti-PD-1 antibody were first examined in the B16 tumor model. C57BL/6 mice were injected with 10^5^ B16 cells subcutaneously on Day 0, and then randomized on Day 3 into four treatment groups (N = 10/group): control; oral opaganib alone (50 mg/kg/day 5 days/week); anti-PD-1 antibody alone (200 µg mouse intraperitoneally on Days 3, 6 and 10); and opaganib +anti-PD-1 antibody. Control group mice received oral vehicle and/or sterile PBS intraperitoneally on all days that treated mice received either opaganib or antibody. Tumors were measured three times per week until mice were euthanized when tumor volumes reached ≥3000 mm^3^. Figure 3 demonstrates that tumors in the control mice grew very aggressively after a lag of approximately 10 days. On Day 19, the average tumor volumes for the control, opaganib alone, anti-PD-1 antibody alone and combination treatment groups were 1702 ± 373, 892 ± 364, 783 ± 265 and 190±114 (*p* = 0.0011) mm^3^, respectively. As directed by the IACUC protocol, each mouse was sacrificed when its tumor volume exceeded 3000 mm^3^. Figure 3 also shows that mice in the control group had a median survival of 21 days, and all animals were sacrificed by Day 29. Treatment with opaganib alone provided a median survival of 24 days and 30% of the mice were alive on Day 56 when the experiment was terminated (*p* = 0.009). Similarly, anti-PD-1 alone enhanced median survival to 23 days (*p* = 0.033) and resulted in 20% of the mice surviving to Day 56. The combination of opaganib plus anti-PD-1 antibody markedly increased median survival to 35 days, and 30% of these mice survived to Day 56 (*p* < 0.0001). Therefore, combining opaganib with anti-PD-1 antibody improves antitumor activity in the B16 tumor model and increases survival longer than does either agent alone.

**Figure 4:**
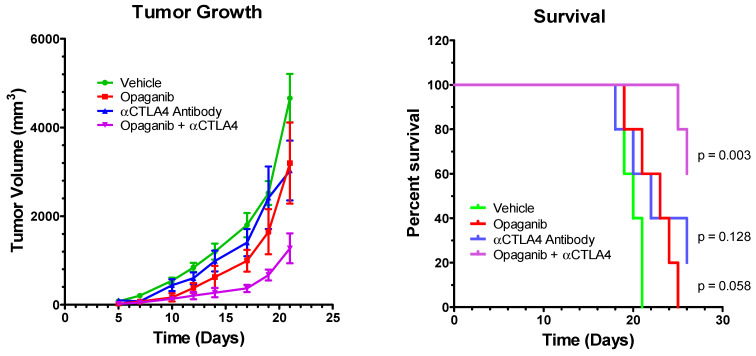
Antitumor activity of opaganib in combination with anti-CTLA-4 antibody. LLC cells were grown as xenografts and treated with the vehicle; opaganib alone (50 mg/kg/day, 5 days/week); anti-CTLA-4 antibody (200 μg/mouse intraperitoneally, 6 times); or opaganib+anti-CTLA-4. (**Left panel**): The mean ± SD tumor volume for each treatment group (n = 5) is shown. (**Right panel**): Individual mice were sacrificed when tumors exceeded 3000 mm^3^ and survival for each group is shown.

Similar to the previous experiment, the combined effects of treating tumor-bearing mice with opaganib and anti-CTLA-4 antibody were examined in the LLC tumor model. Mice were injected with 10^6^ LLC cells into the right hind flank subcutaneously on Day 0 and randomized on Day 3 into four treatment groups (N = 5/group): control; oral opaganib alone (50 mg/kg/day 5 days/week); anti-CTLA-4 antibody alone (200 µg mouse intraperitoneally on Days 3, 6, 10, 13, 17 and 20); and opaganib +anti-CTLA-4 antibody. Control group mice received oral vehicle and/or sterile PBS intraperitoneally on all days that treated mice received either opaganib or antibody. Figure 4 shows that tumors in the control mice grew progressively after a lag of approximately 7 days, and on Day 21, the average tumor volumes for the control, opaganib alone, anti-CTLA-4 antibody alone and combination treatment groups were 4622 ± 548, 3197 ± 914, 3029 ± 675 and 1274 ± 336 (*p* = 0.0008) mm^3^, respectively. Figure 4 also shows that mice in the control group had a median survival of 19 days, and all animals were sacrificed by Day 21. Treatment with opaganib alone provided a median survival of 22 days, while treatment with anti-CTLA-4 did not affect the median survival. The combination of opaganib+anti-CTLA-4 antibody increased median survival to >26 days, and 60% of these mice survived to Day 26. Therefore, combining opaganib with anti-CTLA-4 also provides significantly improved antitumor activity in the LLC tumor model and increases survival longer than does either agent alone.

The combined effects of treating tumor-bearing mice with opaganib and anti-PD-L1 antibody were also examined in the B16 tumor model. Mice were injected with 100,000 B16 cells suspended in PBS into the right hind flank subcutaneously on Day 0 of the experiment. When the tumor reached a volume of ≥300 mm^3^, mice were randomized into the following four treatment groups (n = 5–6/group): control; oral opaganib alone (50 mg/kg/day 5 days/week); anti-PD-L1 antibody alone (200 µg mouse intraperitoneally on Days 1, 3, 5 and 7); and opaganib in combination with anti-PD-L1 antibody. Control group mice received the oral vehicle and/or sterile PBS intraperitoneally on all days that treated mice received either opaganib or antibody. Mice in the control group had a median survival of 8.5 days, and all animals were sacrificed by Day 12 (Table 2). Treatment with opaganib alone or anti-PD-L1 alone provided median survivals of 10 and 10.5 days, respectively (*p* = 0.19 and *p* = 0.2), while combination of opaganib plus anti-PD-L1 antibody increased median survival to 16 days (*p* = 0.0029 compared with control). Therefore, combining opaganib with the PD-L1 checkpoint antibody significantly increases survival longer than does either agent alone in the B16 tumor model.

## 3. Discussion

Sphingolipid metabolism has been the subject of substantial investigation because of its pleotropic roles in cancer biology and response to chemotherapy. In particular, phosphorylation of sphingosine by sphingosine kinases (SK1 and SK2) to produce S1P promotes proliferation and protects against ceramide-induced apoptosis. Mechanistically, SK activity promotes signaling through the Ras/Erk/Akt pathway [40,41,42,43,44,45,46,47,48,49] and regulates the sensitivities of tumor cells to anticancer drugs [50,51]. Therefore, inhibition of SK is expected to increase tumor chemosensitivity by elevating ceramide levels in the cells. Because SK1 and SK2 derive from distinct genes and have different subcellular localizations and biological functions, there has been considerable debate over which isozyme is the best target for new drugs [15,52,53]. We have previously shown using SK inhibitors and RNA interference that basal or induced levels of SK1 (or exogenous S1P) cannot overcome the antiproliferative effects of inhibition of SK2 [23]. Similar lack of functional compensation has also been demonstrated by other groups [54,55,56,57,58], and this likely reflects the different subcellular localizations of S1P produced by SK1 and SK2. Overall, the propensity of data indicates that SK2 is a key mediator of enhanced growth of cancer. In parallel, de novo synthesis of ceramide requires DES1 to introduce the unsaturated C-C double bond into the sphingosine moiety. Inhibition of DES1 elevates dhCer which promotes ER stress and lethal autophagy in cancer cells [59,60,61]. Thus, dual targeting of SK2 and DES1 results in suppression of proliferative signaling and promotion of autophagic cancer cell death.

We identified structurally novel drug-like inhibitors of SK2 and DES1, and opaganib (formerly called ABC294640) was selected as the first investigational new drug for clinical testing [22]. Inhibition of SK2/DES1 by opaganib has been shown to block Akt signaling [22,23,26,27,32], and downregulate the expression of the c-Myc [26,27,28,29,62] and Mcl-1 [28] proteins, resulting in inhibition of proliferation and promotion of tumor cell death. Multiple studies have confirmed the in vivo antitumor activity of opaganib in a wide range of mouse models [15], including breast [22,25,30,63], kidney [31], liver [32,64], colon [34], lung [65], pancreas [66], prostate [26,27], ovary [67], cervix [68], skin [66,69], lymphoma [33], myeloma [28,70] and leukemia [62]. In addition to efficacy as a single agent, opaganib can be safely and effectively combined with other anticancer drugs and/or radiation [24,32]. A Phase 1 trial conducted with patients with advanced solid tumors demonstrated that opaganib is well-tolerated when administered orally on a BID continuous schedule [35]. Overall, 64% of patients who completed two cycles of opaganib treatment had stable disease. In a second clinical trial of opaganib 58% of patients with refractory multiple myeloma achieved stable disease or better, and patients had decreased plasma levels of TNFα, EGF and VEGF [36]. Additional work demonstrated that opaganib has antiviral activity against several viruses including SARS-CoV-2. Therefore, opaganib was evaluated in patients hospitalized with severe COVID-19 pneumonia [71], including a Phase 2a study [72] and a Phase 2/3 multinational randomized, placebo-controlled study. These trials demonstrated the safety of opaganib and a clinical benefit to patients requiring oxygen supplementation of 60% or less (62% reduction in rate of ventilation and death) [73]. To date, more than 470 people have been treated with opaganib in oncology and COVID-19 clinical trials, demonstrating the excellent safety profile of the drug even in severely compromised patients.

While the molecular drivers of ICD remain to be elucidated, it is striking that the cellular processes involved in ICD are known to be regulated by sphingolipid metabolism [74,75,76,77,78,79]. For example, elevation of ceramides enhances ICD in response to mitoxanthrone and photodynamic therapy [80]. SK inhibitors, including opaganib, further elevated ceramide levels, surface expression of calreticulin and release of ATP from mitoxanthrone-treated [37] or ABT-263/AZD-5991-treated [38] colorectal cells. Herein, we demonstrate that an opaganib concentration that can be achieved in vivo [22] promotes calreticulin surface expression of a variety of tumor cell types. Furthermore, the present studies provide in vivo data supporting the potential for improving checkpoint antibody-based cancer immunotherapy by combination with opaganib. In the first series of in vivo studies, the well-established vaccination assay was used to demonstrate the induction of ICD by treatment of tumor cells with opaganib. The data across three independent murine tumor cell lines consistently show that implantation of in vitro opaganib-treated cells suppresses the growth of tumors from subsequently injected tumor cells. One potential use for these findings may be in the ex vivo induction of tumor immunity for cancer patients. Interestingly, ICD promoted immunity against not only the originally administered tumor cells, but also against the alternate murine tumor cells, i.e., cross-immunity. Thus, the ICD-inducing neoantigen(s) expressed following opaganib treatment appear to be conserved across the cell lines used. This may suppress secondary tumors in opaganib-treated cancer patients.

The mechanism by which ICD enhances antitumor activity of checkpoint antibody efficacy is also poorly understood; however, increasing data suggest roles for sphingolipids in this process consistent with the established pharmacologic effects of opaganib. For example, genetic ablation of SK1 slowed melanoma tumor growth and enhanced the antitumor activity of antibodies against CTLA-4 or PD-1 in C57BL/6 mice by increasing the CD8/Treg ratio in the tumors [81]. Parallel studies with inhibition of SK2 were not described. Similar studies by Lau et al. showed that PF543, a dual SK1/SK2 inhibitor with higher potency toward SK1 [82], enhanced the efficacy of anti-PD-1 antibodies against melanoma tumors, possibly by downregulation of MTA3 and c-Myc expression [83]. The current studies lay the groundwork for follow-on analyses of the mechanism by which opaganib modulates antitumor immunity, which may involve intratumoral effects such as modulation of the infiltration of immune cells as discussed above and/or enhancement of T-cell immune responses.

**Figure 5:**
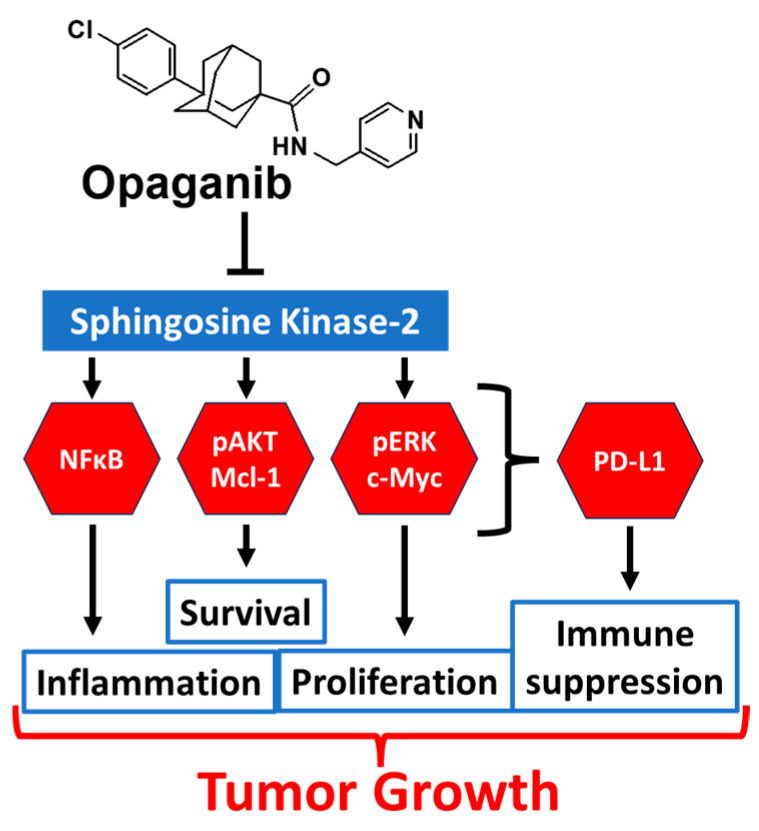
Model for antitumor activity of opaganib. Opaganib acts as a sphingosine-competitive inhibitor of SK2 suppressing signaling through multiple pathways in tumor cells and thereby inhibiting proliferation, survival and inflammation. These pathways are also known to regulate PD-L1 expression allowing opaganib to also suppress antitumor immunity.

Additional evidence suggests that sphingolipids may regulate checkpoint protein expression. For example, PD-L1 expression in tumor cells is induced by IFN-γ [84,85,86,87], and IFNγ signaling is dependent on sphingolipid metabolism [88,89,90,91]. Furthermore, AKT- [92,93,94,95,96,97,98], NFκB- [99], TNFα- [100] and STAT3 [101,102,103] signaling all induce PD-L1 expression, and all of these pathways are downregulated by opaganib. Importantly, several studies demonstrate that c-Myc promotes PD-L1 expression in a variety of tumor types [104,105,106,107,108,109], and anti-PD-L1 and the c-Myc inhibitor JQ1 cause synergistic inhibition of pancreatic cancer xenografts [110]. We and others have shown that opaganib decreases c-Myc expression in many types of cancer cells [26,27,28,29,67,70]. Figure 5 is a mechanistic working model integrating the known signaling effects of opaganib with the potential for downregulation PD-L1 expression on tumor cells.

Regulation of CTLA-4 and PD-1 expression by exhausted T cells is less understood mechanistically. Genetic and biochemical studies have identified at least 10 transcription factors that modulate PD-1 expression, including NFAT2, AP-1 and STAT3 [111]. Regulation of AP-1 by sphingolipids has been shown in many systems (reviewed in [112]), and opaganib inhibits STAT3 activation [113]. The majority (~90%) of CTLA-4 protein in resting T cells is localized in endocytic vesicles and so increased expression at the cell surface is likely regulated by pathways that control vesicle trafficking [114,115]. Membrane dynamics and vesicle trafficking are also modulated by sphingolipid metabolism (reviewed in [116,117]). Therefore, it is reasonable to predict that alteration of the sphingolipid profile in response to opaganib treatment may alter the surface expression of PD-1 and/or CTLA-4.

Overall, the studies herein demonstrate the ability of opaganib to induce ICD, in which dying tumor cells promote immunologic responses that enhanced the in vivo activity of checkpoint antibodies in multiple tumor types. This supports consideration of the use of opaganib in combination with current checkpoint antibodies in clinical trials against lung cancer, melanoma, neuroblastoma and potentially other cancer types.

## 4. Materials and Methods

### 4.1. Materials

Tumor cell lines B16 melanoma, Lewis lung carcinoma (LLC), Neuro-2a, PAN02, TRAMP-C2 and E0771 were purchased from the American Type Culture Collection (Manassas, VA, USA). Opaganib (GMP-grade) was synthesized according to French et al. [22] and dissolved in a vehicle consisting of 46.7% polyethylene glycol 400, 46.7% saline and 6.6% EtOH. Anti-mouse PD-1 antibody (Catalog number BE0146), anti-mouse PD-L1 (catalog number BE0101) and anti-mouse CTLA-4 antibody (Catalog number BE0131) were purchased from BioXCell (West Lebanon, NH, USA) and suspended in sterile PBS for intraperitoneal administration.

### 4.2. In Vitro Calreticulin Surface Expression Assay

Cultures of PAN02, TRAMP-C2, Neuro-2a, E0771, LLC and B16 cells were grown in 10 cm dishes to approximately 50% confluency. Cells were then treated with 0 or 40 µM opaganib for 24 h, harvested by trypsination, washed and resuspended in PBS. Cells were stained with phycoerythrin-conjugated anti-calreticulin antibody (Cell Signaling Technology, Beverly, MA, USA) at 4 °C for 1 h and analyzed by flow cytometry (BD Biosciences, San Jose, CA, USA) in the Penn State Cancer Institute Shared Resource.

### 4.3. In Vivo Vaccination Assay

Murine melanoma B16, lung LLC or Neuro-2a neuroblastoma cells were treated in vitro with 40 μM opaganib for 24 h. The cells were then washed, harvested, and suspended in PBS. The opaganib-treated cells were implanted subcutaneously into the left hind flank of otherwise untreated C57BL/6 (5 × 10^5^ B16 cells/mouse or 5 × 10^6^ LLC cells/mouse) or A/J (5 × 10^6^ Neuro-2a cells/mouse) mice. Control groups were injected in the left hind flank with PBS alone. After 7 days, mice were implanted with 1/5 the number of untreated matching tumor cells on the right hind flank, and tumor growth was measured until mice were euthanized when tumor volumes reached ≥3000 mm^3^.

### 4.4. In Vivo Tumor Growth Assays

C57BL/6 mice were injected with 10^5^ B16 cells suspended in PBS into the right hind flank subcutaneously on Day 0 of the experiment. Mice were randomized on Day 3 into the following four treatment groups (n = 5–10/group): control (vehicle only); opaganib alone (oral gavage at 50 mg/kg 5 days/week until sacrifice); antibody alone (intraperitoneal injection at a dose of 200 µg/mouse on days indicated in the specific experiments); and opaganib in combination with antibody. The combination treatment group mice received antibody and opaganib treatments concomitantly on days when antibody was scheduled. Tumors were measured with digital calipers three times per week and volumes were calculated using the formula V= (L × W2)/2. Toxicity of the treatments was assessed by careful observation of the mice for signs of distress, including respiratory difficulties, gastrointestinal distress, evidence of spastic paralysis, convulsion, or blindness. No mice displayed any of these abnormalities, so individual mice were euthanized by CO_2_ asphyxiation and cervical dislocation when the tumor volume exceeded ≥3000 mm^3^.

### 4.5. Statistics

Mouse survival rates were compared using the Kaplan–Meier approach with the Gehan–Breslow–Wilcoxon test using GraphPad Prism software (Version 5.00). Other data were analyzed by one-way ANOVA using the Tukey post hoc test. Differences were considered to be statistically significant when *p* < 0.05. Error bars in the figures represent the mean ± standard deviation of the treatment groups calculated with GraphPad Prism.

## Figures and Tables

**Table 1:** Effects of opaganib on calreticulin cell surface expression. Each cell type was treated with 0 or 40 µM opaganib for 24 h. The geometric mean of the fluorescence intensity for cells treated with the vehicle is expressed as 1.0 and samples treated with opaganib are expressed relative to the vehicle control.

Tissue	Cell Line	Surface Calreticulin Expression (Geometric Mean)	
		Control	Opaganib (40 µM)
Pancreas	PAN02	1.0	2.31
Prostate	TRAMP-C2	1.0	3.64
Neuroblastoma	Neuro-2a	1.0	3.0
Breast	E0771	1.0	1.46
Lung	LLC	1.0	3.0
Melanoma	B16	1.0	2.7

**Table 2:** Antitumor activity of opaganib in combination with anti-PD-L1 antibody. B16 cells were grown as xenografts and treated with the vehicle; opaganib alone (50 mg/kg/day, 5 days/week); anti-PD-L1 antibody (200 μg/mouse intraperitoneally, 3 times); or opaganib+anti-PD-L1. Individual mice were sacrificed when tumors exceeded 3000 mm^3^ and survival for each group is shown.

Group	Treatment	Number of Mice	Median Survival (Days)	Significance Compared with Control (*p*)
1	Vehicle	6	8.5	
2	Opaganib alone	5	10	0.19
3	Anti-PD-L1 antibody alone	6	10.5	0.2
4	Opaganib plus anti-PD-L1 antibody	5	16	0.0029

## Data Availability

Data is contained within the article.
